# Comment on: “Power Training Prescription in Older Individuals: Is it Safe and Effective to Promote Neuromuscular Functional Improvements?”

**DOI:** 10.1007/s40279-023-01919-9

**Published:** 2023-09-14

**Authors:** Ronald E. Michalak

**Affiliations:** Peterborough, NH USA

Dear Editor,

The recent article by Radaelli et al. [1] presents a compelling argument that power training is safe, effective, and necessary for aging adults and should be regarded as a call to action to give this critical issue the attention that it deserves. Recently presented data on single muscle fiber contractile function with aging highlighted the preferential decline in type II fibers [2]. This offers underlying mechanistic support for the critical need for power training to prevent functional decline with aging.

Radaelli et al. [1] then pose the appropriate question: ‘Why is the loss of power and power training overlooked by health professionals and many international organizations?’

The simple answer is that loss of power does not yet have a name.

*Sarcopenia* is generally considered a loss of muscle mass. A quick literature search revealed the first four mentions of sarcopenia in 1993 [3–6]. Since 1993, there have been 17,184 additional references to sarcopenia. Since having a name, sarcopenia has garnered research and clinical attention. However, sarcopenia still lacks consensus on clear diagnostic criteria [7].

The first reference to *dynapenia* that I could locate was by Clark and Manini in 2008 [8], who made the case that *dynapenia* (loss of strength) is a related but separate quality to *sarcopenia*, loss of muscle mass. Since 2008, there have been 359 additional references to *dynapenia*. Since having a name, *dynapenia* has received attention in research, but less so clinically. Some of the difficulty lies in definitions. For instance, grip strength, which is an appropriate measure of strength for diagnosis of *dynapenia*, is sometimes used as a diagnostic criterion for *sarcopenia* [7]. To confuse matters even further, walking speed, which is more related to power than to strength or muscle mass, is also used as a diagnostic criterion for *sarcopenia* [7].

Contemporary block periodized training for athletes is based upon differential training stimuli for muscle mass (hypertrophy), maximal strength, and power [9]. These concepts are not new and the differential effects of heavy versus explosive exercise were noted over 800 years ago by Abul-Walid Ibn Rushd (1126–1198 CE) [9]. These three separate qualities deserve attention in the aging population as well as in athletes.

Radaelli et al. [1] made a clear case that *loss of power* is the enemy of aging and is more strongly associated with functional measures than muscle mass or even strength. It deserves attention both in research and in clinical application. I believe that it has been overlooked clinically as it does not yet have a name.

Name the enemy. A name allows us to define it. A name allows us to categorize it. A name allows us to find the best ways to defeat it. Moreover, a name is a rallying cry for action.

How to name it? First, look up the definition of ‘power’ in the physics section of Wikipedia [10]. Then, use the translate function on this website to see that ‘potentia’ is the Latin translation of the word power. Therefore, I would propose calling the loss of muscular power *potentiapenia*.

Proposed operational definitions:

- *Sarcopenia* Loss of muscle mass
- *Dynapenia* Loss of muscular strength
- *Potentiapenia* Loss of muscular power

Having three specific names for three related, but clearly distinct, qualities may assist the international community in defining these issues. It will take time and effort to develop consensus. However, this will promote consistent and comparable research as well as clinical application for the betterment of the aging worldwide population.

In 1993, Dr. Butler, the editor-in-chief of the journal *Geriatrics*, stated "Is there anything we can do to promote good health and disease prevention? Perhaps we could invent a new disorder of aging… because [people] are famous for responding to identified diseases… A new, well publicized disorder… might stimulate a… public response" [4].

As long as 30 years ago, in 1993, Rogers and Evans [6] mentioned preferential atrophy of type II fibers with aging and stated that muscular functional decline associated with aging "can no longer be considered as an inevitable consequence of the aging process." Unfortunately, not enough clinical attention has been directed towards this issue over the last 30 years. It is time to address *potentiapenia*.
